# Does intraspecific variation in juvenile Late Cretaceous ammonoids correlate with their systematic position, longevity and paleogeography?

**DOI:** 10.1186/s13358-025-00397-y

**Published:** 2025-08-19

**Authors:** Amane Tajika, Takahiro Iida, Ryoji Wani, Neil H. Landman, Kenji Ikuno, Christian Klug

**Affiliations:** 1 Hakubi Center for Advanced Research, Kyoto University, Yoshida-Honmachi, Sakyo-Ku, Kyoto, 606-8501 Japan; 2 Graduate School of Human and Environmental Studies, Kyoto University, Yoshida Nihonmatsu-Cho, Sakyo-Ku, Kyoto, 606-8316 Japan; 3 Division of Paleontology (Invertebrates), American Museum of Natural History, Central Park West 79th Street, New York, NY 10024 USA; 4 Graduate School of Environment and Information Sciences, Yokohama National University, Yokohama, 240‑8501 Japan; 5 Faculty of Environment and Information Sciences, Yokohama National University, Yokohama, 240‑8501 Japan; 6 Division of Natural History, Museum of Nature and Human Activities, Sanda, Hyogo 669-1546 Japan; 7 Division of Earth Sciences, Institute of Natural and Environmental Sciences, University of Hyogo, Sanda, 669-1546 Japan; 8 Paläontologisches Institut, Universität Zürich, Karl-Schmid-Strasse 4, 8006 Zurich, Switzerland

## Abstract

Phenotypic intraspecific variation of organisms is essential for evolution and, thus, has the potential to provide crucial insights into evolutionary dynamics. Additionally, ontogeny is often intricately linked with the evolutionary trajectories of organisms. In this study, we explore the relationship between the magnitude and ontogenetic pattern of intraspecific variation, and the interrelationships of organisms, their geographic distribution, and species duration. We analyzed the intraspecific variation in the whorl expansion rate (WER) of several Late Cretaceous ammonoid species, including *Hypophylloceras ramosum*, *Phyllopachyceras ezoense*, *Gaudryceras tenuiliratum*, *Tetragonites glabrus*, *T. popetensis*, *Damesites damesi*, *Tragodesmoceroides subcostatus*, *Subprionocyclus minimus*, *Yezoites puerculus* (all from Hokkaido, Japan), as well as *Scaphites whitfieldi* and *Hoploscaphites comprimus* (both from North America). Our results reveal a weak to moderate, negative correlation between the magnitude of intraspecific variation and geographic distribution. The correlation between intraspecific variation and species duration is weak. Notably, scaphitid and phylloceratid ammonoids exhibit a higher degree of intraspecific variation compared to other species, although no significant differences are apparent within each family. Additionally, scaphitid species from both Japan and North America display similar ontogenetic patterns of intraspecific variation. *Hypophylloceras ramosum* exhibits a pattern of intraspecific variation, differing from other normally coiled ammonoids. In other taxa, the pattern among species cannot be distinguished. These discoveries suggest that intraspecific variation, geographic distribution, and species duration are, at least, not positively correlated. However, contrary to previous studies, our data suggest a potential link between intraspecific variation and the interrelationships of species (relative phylogenetic position). Further research involving the analysis of more taxa, multiple morphological parameters examined over longer ontogenetic stages, and the development of a robust phylogenetic hypothesis are necessary to better understand these associations.

**Supplementary Information:**

The online version contains supplementary material available at 10.1186/s13358-025-00397-y.

## Introduction

Since the fundamental work of Wallace (1858) and Darwin (1859), it has been widely recognized that phenotypic variation of organisms plays an important role in their evolution. Subsequent studies demonstrated that phenotypic intraspecific variation is generated by various factors, including genetic diversity, adaptation, and phenotypic plasticity, which stimulated researchers to examine various aspects of intraspecific variation. An increasing number of studies highlight the impact of intraspecific variation on ecology and evolution. For instance, Palkovacs and Post (2009) demonstrated that increased variation in gill raker spacing in alewife fish caused a decrease in body size and diversity of zooplankton that the fish filtered. Similarly, Des Roches et al. (2013) observed that phenotypic diversity in stickleback fish significantly influenced the community structure at lower trophic levels. In paleontology, intraspecific variation is recognized as an important parameter for taxonomic work. Yet, few studies have attempted to link species evolvability and intraspecific variation. For example, Liow (2007) discovered that in trachyleberidid ostracods, morphological intraspecific variation of species and genera bearing subspecies and species with high variation exhibits a longer geological duration (taxon longevity). In contrast, Hopkins (2011) reported no positive correlation between morphological intraspecific variation and geological duration, and geographical distribution in late Cambrian trilobites, nor did her study detect a phylogenetic signal with respect to intraspecific variation. Additionally, Hunt (2007) found that evolutionary changes in species occurred in the direction of higher morphological variation.

Ectocochleate cephalopods such as ammonoids and nautiloids were one of the most abundant invertebrates that thrived in ancient oceans for hundreds of millions of years. Because of their high abundance, high evolutionary rates, and worldwide distribution, they are frequently employed as model organisms to test macroevolutionary hypotheses. Examining and understanding morphological intraspecific variation is essential for taxonomic work, which, in turn, is crucial for precise species counts in cephalopods, especially in ammonoids (e.g., Bert et al., 2013; Callomon, 1985; De Baets et al., 2013; Jattiot et al., 2021; Klug, 2017; Korn, 2017; Landman et al., 2010; Rogov & Kiselev, 2024; Shigeta, 1989; Weinkauf et al., 2021). Furthermore, researchers have explored the relationship between morphological changes within species (Monnet et al., 2015) and abiotic factors such as bathymetry (Kawabe, 2003; Landman & Waage, 1993a; Wilmsen & Mosavinia, 2011).

One significant advantage of studying ectocochleate cephalopods is the accretionary growth of their shell, which allows for the detailed examination of ontogeny within an individual. Recent studies have shown that intraspecific variation changes during ontogeny (Bersac & Bert, 2012; De Baets et al., 2013; Klein & Landman, 2019; Korn & Klug, 2007; Tajika et al., 2018, 2020). However, little is known pertaining to the underlying biological mechanisms for varying degrees of variation during ontogeny (Urdy et al., 2010). Given that the magnitude of intraspecific variation is likely linked to the adaptability of organisms, it may have evolutionary implications. Despite this, the link between intraspecific variation and the evolutionary dynamics in ectocochleate cephalopods has been relatively underexplored. To enhance our understanding of the roles of intraspecific variation in cephalopod evolution, it is essential to comparatively examine multiple taxa. Early ontogeny, during which key life events occur (e.g., hatching and potentially the transition from a planktic to a nektobenthic, nektic or nektoplanktic lifestyle), may provide critical insights into the relationship between intraspecific variation and evolutionary dynamics. In this study, we investigate 11 Late Cretaceous ammonoid species with a focus on the whorl expansion rate and early ontogeny and aim to answer the following questions: (1) Is there a correlation between the magnitude of intraspecific variation during early ontogeny and the species duration or geographic distribution? (2) Does the ontogenetic pattern of intraspecific variation among species reflect species duration and geographic distribution? (3) Do the magnitude and pattern of intraspecific variation reflect the interrelationships of species?

## Material and methods

**Fig. 1:**
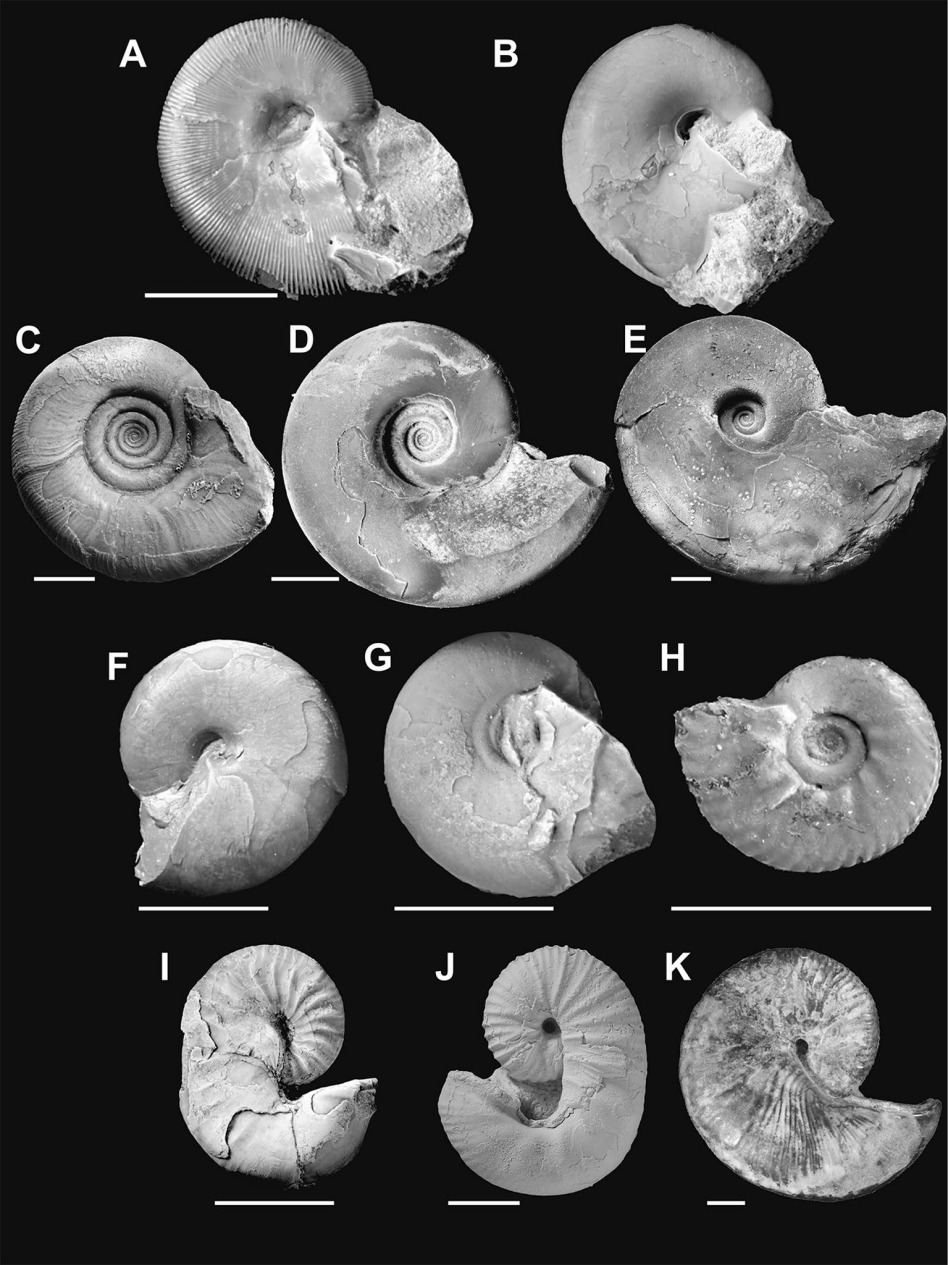
Studied ammonoid species. **A** *Hypophylloceras ramosum* from the Campanian of Hokkaido, Japan (MCM-W1771). **B** *Phyllopachyceras ezoense* from the Campanian of Hokkaido, Japan (MCM-W1827). **C** *Gaudryceras tenuiliratum* from the Campanian of Hokkaido, Japan (MCM-W1869). **D** *Tetragonites popetensis* from the Campanian of Hokkaido, Japan (MCM-W2010). **E** *T. glabrus* from the Santonian of Hokkaido, Japan (MCM-W1991). **F** *Damesites damesi* from the Santonian of Hokkaido, Japan (MCM-W1103). **G** *Tragodesmoceroides subcostatus* from the Turonian of Hokkaido, Japan (MCM-W1906). **H** *Subprionocyclus minimus* from the Turonian of Hokkaido, Japan (MCM-W2103). **I** *Yezoites puerculus* from the Turonian of Hokkaido, Japan (MCM-W1307). **J** *Scaphites whitfieldi* from the Turonian of South Dakota, USA (AMNH 82694). **K** *Hoploscaphites comprimus* from the Maastrichtian of South Dakota, USA (YPM 27234). *MCM* Mikasa City Museum, *AMNH* American Museum of Natural History. Scale bars = 10 mm

**Fig. 2:**
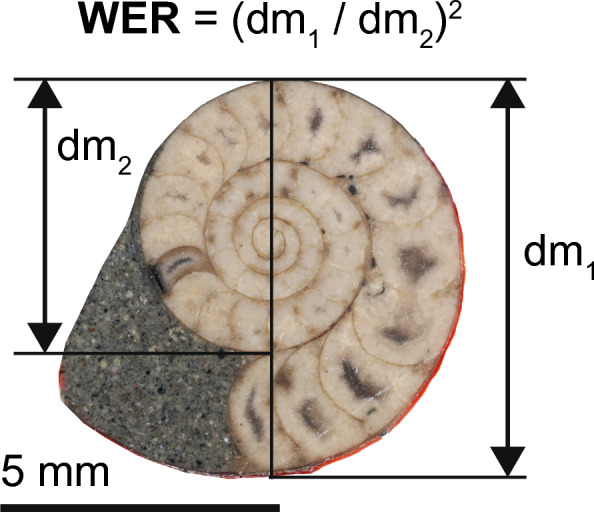
Conch parameters measured in this study (*Yezoites puerculus* from the Turonian of Hokkaido; MCM-W 2151). *dm* conch diameter. *WER* whorl expansion rate

In this paper, we follow the classification of Wright et al. (1996). We studied a total of 462 specimens in 11 ammonoid species representing six families and four suborders from the Late Cretaceous of Japan and North America (Fig. 1; Table 1; Supplemental Table)—*Hypophylloceras ramosum* (Meek, 1858) from the Santonian and Campanian, *Phyllopachyceras ezoense* (Yokoyama, 1890) from the Campanian, *Gaudryceras tenuiliratum* Yabe, 1903 from the Santonian and Campanian, *Tetragonites glabrus* (Jimbo, 1894) from the Santonian, *T. popetensis* Yabe, 1903 from the Campanian, *Damesites damesi* (Jimbo, 1894) from the Santonian and Campanian, *Tragodesmoceroides subcostatus* Matsumoto, 1942 from the Turonian*, Subprionocyclus minimus* (Hayasaka & Fukada, 1951) from the Turonian, *Yezoites puerculus* (Jimbo, 1894) from the Turonian (all from Hokkaido, Japan), *Scaphites whitfieldi* Cobban, 1951 from the Turonian (from Colorado, Montana, South Dakota, and Utah of USA), and *Hoploscaphites comprimus* (Owen, 1852) from the Maastrichtian (from South Dakota, USA). Most specimens were previously examined by Tanabe and Shigeta (1987), Landman and Waagé (1993b), Tanabe et al. (2003), Yahada and Wani (2013), Klein and Landman (2019), Iwasaki et al. (2020), Kawakami et al. (2022), Takai et al. (2022), Kawakami and Wani (2023), and Nishino et al. (2024). Some additional specimens from Hokkaido were also examined. Note that we consider *Hy. subramosum*, designated by Iwasaki et al. (2020), as *Hy. ramosum*, following Shigeta et al. (2019). All specimens were either polished to the median section (i.e., the plane of symmetry) or to the plane perpendicular to the plane of symmetry. The median section was photographed to measure the conch diameters (dm) through ontogeny (Fig. 2). Using the measurements, we calculated the whorl expansion rate [WER; (dm_1_/dm_2_)^2^]. Because most specimens are housed in museum collections, we were unable to conduct further destructive analyses.

**Table 1:** Examined ammonoid species

Species	Suborder	Family	Stage	Locality	Number of specimens	References
*Hypophylloceras ramosum*	Phylloceratina	Phylloceratidae	Santonian	Hokkaido, Japan	44	Iwasaki et al. (2020)
			Campanian	Hokkaido, Japan	35	Iwasaki et al. (2020)
*Phyllopachyceras ezoense*			Campanian	Hokkaido, Japan	28	Iwasaki et al. (2020)
*Gaudryceras tenuiliratum*	Lytoceratina	Gaudryceratidae	Santonian	Hokkaido, Japan	19	Kawakami et al. (2022)
			Campanian	Hokkaido, Japan	41	Kawakami et al. (2022)
*Tetragonites glabrus*		Tetragonitidae	Santonian	Hokkaido, Japan	20	Kawakami and Wani (2023)
*Tetragonites popetensis*			Campanian	Hokkaido, Japan	21	Kawakami and Wani (2023); Tanabe et al. (2003)
*Damesites damesi*	Ammonitina	Desmoceratidae	Santonian	Hokkaido, Japan	54	Takai et al. (2022)
			Campanian	Hokkaido, Japan	28	Takai et al. (2022)
*Tragodesmoceroides subcostatus*			Turonian	Hokkaido, Japan	14	Takai et al. (2022)
*Subprionocyclus minimus*		Collignoniceratidae	Turonian	Hokkaido, Japan	46	Tanabe and Shigeta (1987); Nishino et al. (2024)
*Yezoites puerculus*	Ancyloceratina	Scaphitidae	Turonian	Hokkaido, Japan	34	Yahada and Wani (2013); Nishino et al. (2024)
*Scaphites whitfieldi*			Turonian	Colorado, Montana, Utah, South Dakota, USA	35	Klein and Landman (2019)
*Hoploscaphites comprimus*			Maastrichtian	South Dakota, USA	43	Landman and Waage (1993a, 1993b)

We calculated equidistant points for WER with a distance of 0.5 mm using linear interpolation. The resultant equidistant points in the same size classes allowed for the calculation of intraspecific variation through ontogeny. We calculated the standard deviation as an index of intraspecific variation for each size class, in which the sample size is > 10. To test for differences in the degree of intraspecific variation among species, we conducted an analysis of variance (Welch’s ANOVA), followed by multiple comparison tests to identify which pairs of taxa showed significant differences. To evaluate the similarity in the ontogenetic pattern of intraspecific variation (i.e., how closely any two species share the shape of their ontogenetic trajectories), we calculated dynamic time-warping (DTW) distances (Meert et al., 2020) in which a lower value indicates a higher similarity. Subsequently, we assembled the resulting pairwise distances into a distance matrix to produce a dendrogram using hierarchical clustering. These statistical tests were performed for a conch diameter of 2.5–9.5 mm. This size range was chosen because it is the interval in early ontogeny in which all species are represented, while providing the widest possible span for comparison. The data of geographic distribution were obtained from the Paleobiology Database (PBDB). Because no/little data for *P*. *ezoense* and *T*. *subcostatus* were available in the PBDB, we sourced locality information from the literature (Iwasaki et al., 2020; Kodama et al., 2002; Obata et al., 1972; Shigeta et al., 1999; Yazykova et al., 2004) and calculated the corresponding paleocoordinates. Using the paleocoordinates, we determined the geographical distribution (convex hull; km^2^). These calculations were carried out using the palaeoverse package in R (Jones et al., 2023). The local species duration in ammonoids from Hokkaido was estimated using stratigraphic occurrences reported by Toshimitsu and Hirano (2000). The global species duration for each species was also calculated using the PBDB. To investigate the associations among intraspecific variation, species duration, and geographical distribution, Spearman’s rank correlations were calculated using the mean value of standard deviation. Welch’s ANOVA was performed with the Statistics and Machine Learning Toolbox in MATLAB (MathWorks) and other calculations were carried out with Python 3.11.7 and the SciPy and DTAIDistance libraries.

## Results

**Fig. 3:**
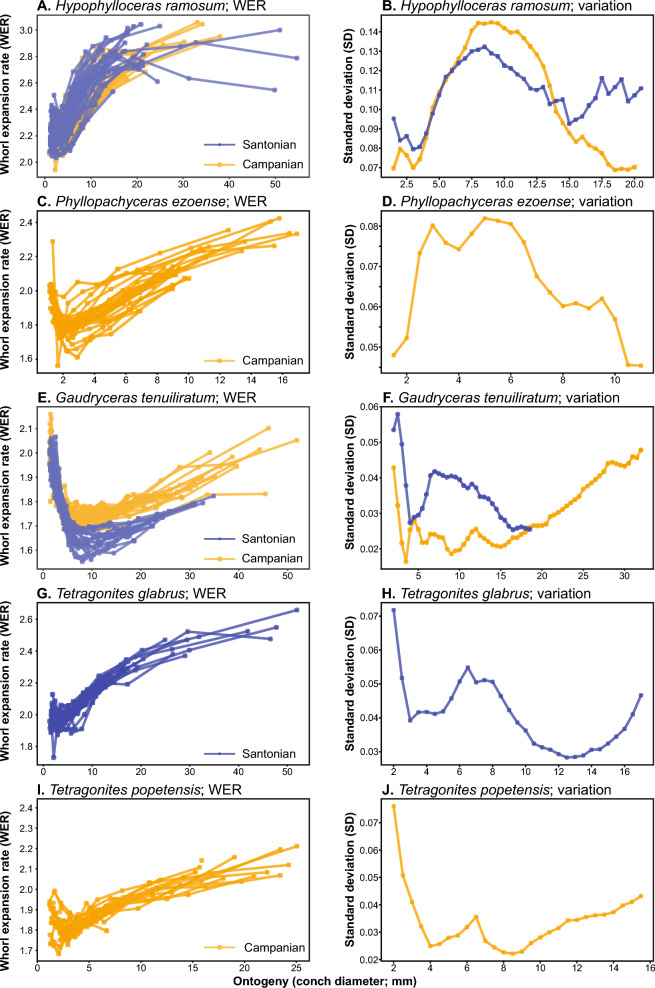
Whorl expansion rate (WER) and the corresponding intraspecific variation through ontogeny in Phylloceratidae (Phylloceratina), Gaudryceratida, and Tetragonitidae (both Lytoceratina). **A** WER in *Hypophylloceras ramosum* from the Santonian and Campanian of Hokkaido, Japan. **B** Intraspecific variation (standard deviation) in *Hypophylloceras ramosum* from the Santonian and Campanian of Hokkaido, Japan. **C** WER in *Phyllopachyceras ezoense* from the Campanian of Hokkaido, Japan. **D** Intraspecific variation (standard deviation) in *Phyllopachyceras ezoense* from the Campanian of Hokkaido, Japan. **E** WER in *Gaudryceras tenuiliratum* from the Santonian and Campanian of Hokkaido, Japan, **F** Intraspecific variation (standard deviation) in *Gaudryceras tenuiliratum* from the Santonian and Campanian of Hokkaido, Japan. **G** WER in *Tetragonites glabrus* from the Santonian of Hokkaido, Japan, **H** Intraspecific variation (standard deviation) in *Tetragonites glabrus* from the Santonian of Hokkaido, Japan. **I** WER in *Tetragonites popetensis* from the Campanian of Hokkaido, Japan. **J** Intraspecific variation (standard deviation) in *Tetragonites popetensis* from the Campanian of Hokkaido, Japan. Geologic stages are colored as follows: Santonian (blue), and Campanian (orange)

**Fig. 4:**
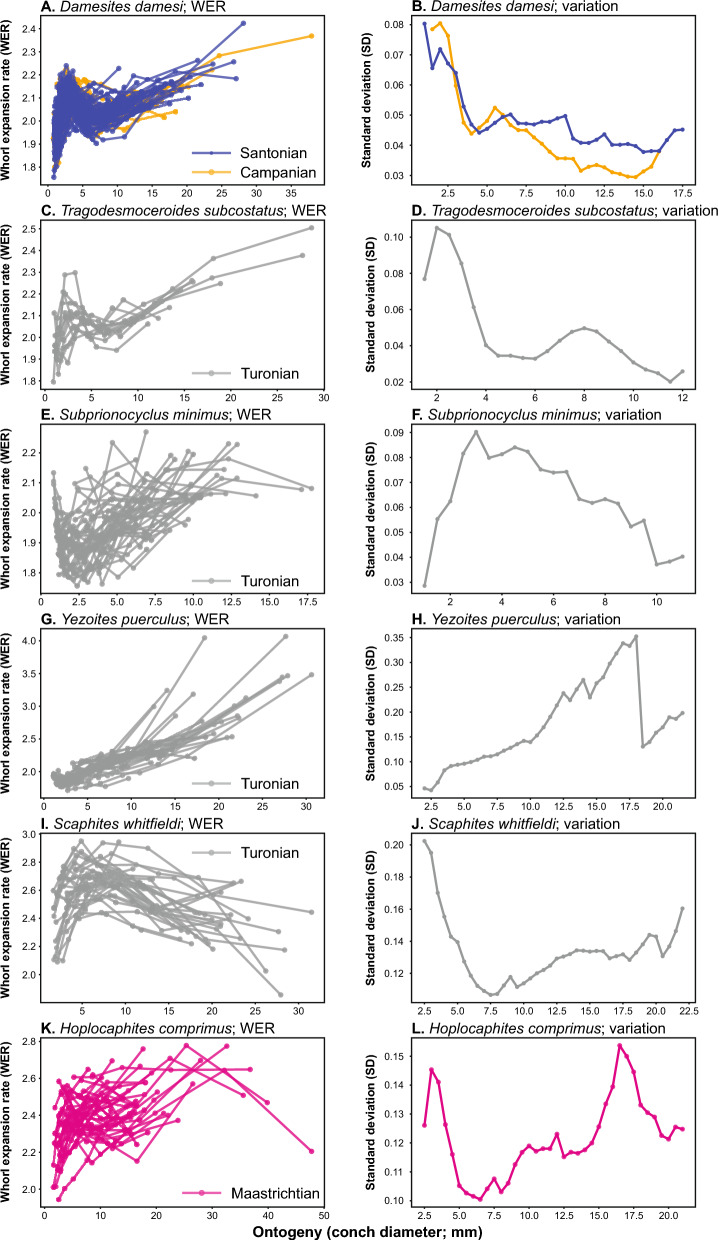
Whorl expansion rate (WER) and the corresponding intraspecific variation through ontogeny in Desmoceratidae, Collignoniceratidae (both Ammonitina), and Scaphitidae (Ancyloceratina). **A** WER in *Damesites damesi* from the Santonian and Campanian of Hokkaido, Japan. **B** Intraspecific variation (standard deviation) in *Damesites damesi* from the Santonian and Campanian of Hokkaido, Japan. **C** WER in *Tragodesmoceroides subcostatus* from the Turonian of Hokkaido, Japan. **D** Intraspecific variation (standard deviation) in *Tragodesmoceroides subcostatus*from the Turonian of Hokkaido, Japan. **E** WER in *Subprionocyclus minimus* from the Turonian of Hokkaido, Japan, **F** Intraspecific variation (standard deviation) in *Subprionocyclus minimus* from the Turonian of Hokkaido, Japan. **G** WER in *Yezoites puerculus* from the Turonian of Hokkaido, Japan, **H** Intraspecific variation (standard deviation) in *Yezoites puerculus* from the Turonian of Hokkaido, Japan. **I** WER in *Scaphites whitfieldi* from the Turonian of Colorado, Montana, South Dakota, and Utah. **J** Intraspecific variation (standard deviation) in *Scaphites whitfieldi* from the Turonian of South Dakota, US. **K** WER in *Hoplocaphites comprimus* from the Maastrichtian of South Dakota, US. **L** Intraspecific variation (standard deviation) in *Hoplocaphites comprimus* from the Maastrichtian of South Dakota, US. Geologic stages are colored as follows: Turonian (gray), Santonian (blue), Campanian (orange), and Maastrichtian (pink)

**Fig. 5:**
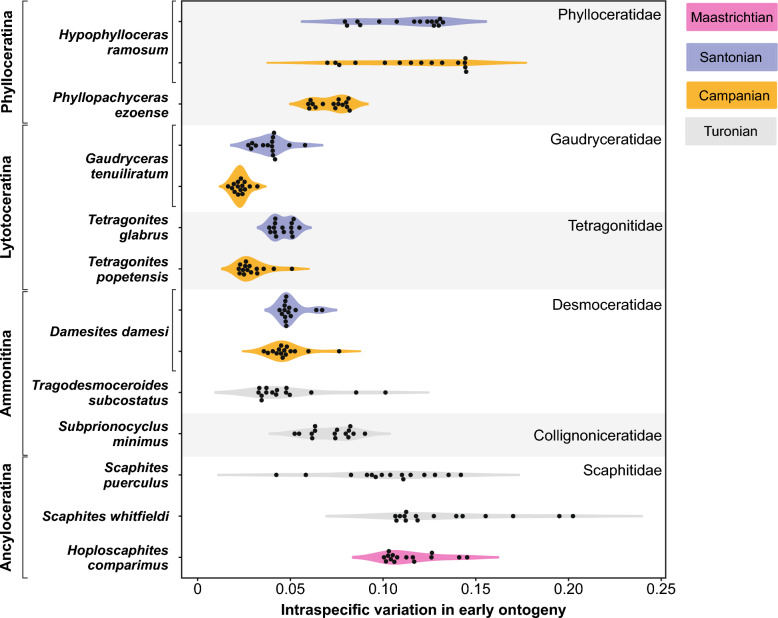
Distribution of intraspecific variation (standard deviation) for the conch diameter of 2.5–9.5 mm. The colored violin plots depict the smoothed distribution (kernel-density estimate); width reflects data density

**Fig. 6:**
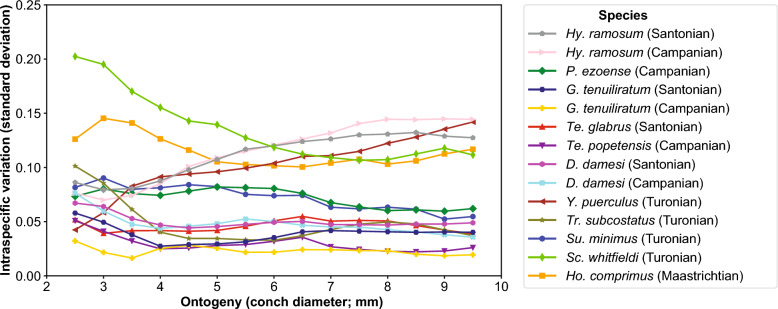
Intraspecific variation for a conch diameter of 2.5–9.5 mm. The pattern of variation in this range was compared among species

**Fig. 7:**
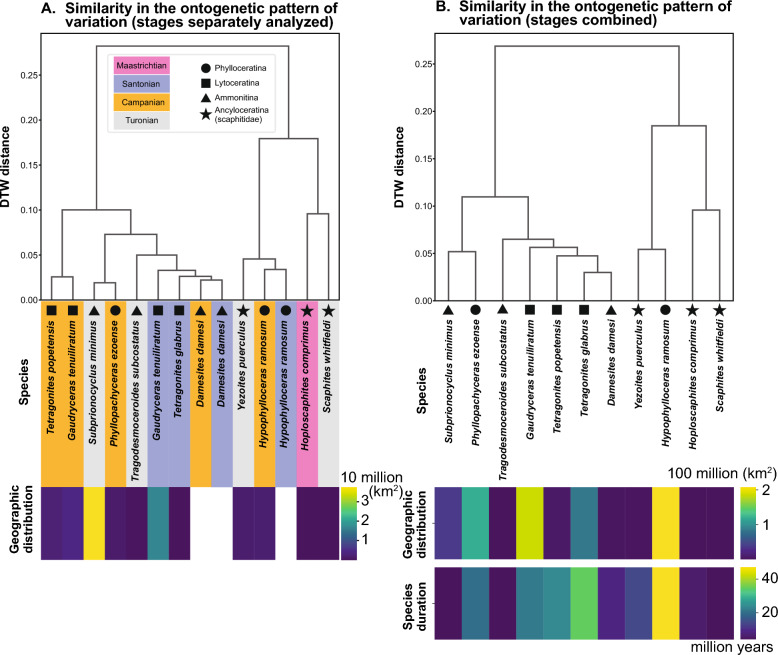
Similarity in the ontogenetic pattern of intraspecific variation among species. Distances were calculated using dynamic time warping (DTW). **A** dendrogram produced using data of intraspecific variation. The Santonian and Campanian specimens of *Gaudryceras tenuiliratum*, *Hypophylloceras ramosum*, and *Damesites damesi* were analyzed separately. Heatmap of the geographic distribution of species in each stage. No data is available for *H. ramosum* from the Camnaian and *D. damesi*. **B** dendrogram produced using data of intraspecific variation. The Santonian and Campanian specimens of *Gaudryceras tenuiliratum*, *Hypophylloceras ramosum*, and *Damesites damesi* were analyzed together. Heatmaps of global geographic distribution and species duration

**Fig. 8:**
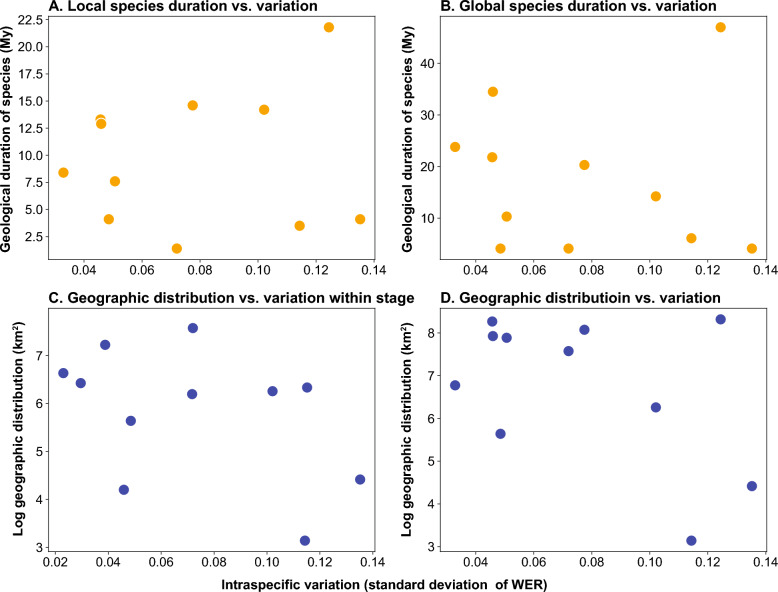
Magnitude of intraspecific variation vs. **A** local species duration, **B**, global species duration, **C** geographic distribution within stage, and **D** geographic distribution for the entire duration of species

The calculated whorl expansion rates (WERs) and intraspecific variation through ontogeny are presented in Figs. 3, 4, and Supplemental Table. The data reveal that different taxa exhibit distinctive patterns of ontogenetic trajectories in WER and the associated intraspecific variation. The distribution of intraspecific variation (standard deviation) for the diameter from 2.5 to 9.5 mm in each species is shown in Fig. 5. A statistical test (ANOVA) indicated a significant difference among species (Table 2). Multiple comparison tests revealed that there is no significant difference within species (*Hypophylloceras ramosum*, *Gaudryceras tenuiliratum*, and *Damesites damesi*) between the Santonian and Campanian (Supplemental Table). Additionally, scaphitid and phylloceratid ammonoids exhibit a magnitude of intraspecific variation higher than those of desmoceratid, tetragonitid, and collignoniceratid ammonoids (Fig. 5). The similarity among species in the ontogenetic pattern of intraspecific variation for the conch diameter range of 2.5–9.5 mm (Fig. 6) was visualized in a dendrogram using hierarchical clustering (Fig. 7). The results reveal that scaphitid species cluster together, whereas other taxa including lytoceratids, desmoceratids, collignoniceratids, and phylloceratids are less distinct and appear indistinguishable. We plotted species duration, geographic distribution, and intraspecific variation (the mean value of standard deviation) in Fig. 8. Although there is a tendency for geographic distribution to decrease with increasing intraspecific variation, the trend is not statistically significant (Table 3). Additionally, the correlation between taxon duration and intraspecific variation is weak.

**Table 2:** Result of Welch’s ANOVA for the magnitude of intraspecific variation among species for the conch diameter of 2.5–9.5 mm

	Sum of squares	Degree of freedom	Mean squares	*F*-statistic	*p*-value
Between groups	0.27	13	0.02	70.78	< 0.000001
Within groups	0.057	196	0.00029		
Total	0.32	209

**Table 3:** Spearman’s rank correlations between the magnitude of intraspecific variation, geological distribution, and species duration

	Spearman's correlation coefficient	*p*-value
Degree of variation of species versus		
local species duration	0.0046	0.99
global species duration	–0.28	0.40
geographic distribution	–0.28	0.40
Degree of variation within stage versus		
geographic distribution within stage	–0.42	0.20

## Discussion

### Biases

Fossil assemblages are unlikely to represent a single population due to various inherent biases. One significant factor is the difference in the magnitude of time-averaging among the studied ammonoid assemblages, which could influence the degree and pattern of intraspecific variation (De Baets et al., 2015). Additionally, sampling intensity within a given time bin is never consistent across species, and sample sizes are also not uniform. These biases are inevitable in the study of fossil groups. In our dataset, for instance, the number of specimens per species ranges from 14 to 54 (Table 1), which may mask the actual pattern of intraspecific variation. Another potential source of bias is post-mortem transport, which may lead to the mixing of multiple contemporaneous populations.

Despite these potential biases, we believe that our results reflect biological signals, based on the following assumptions: (1) the local pattern of intraspecific variation more or less implies the global pattern because we examined each species from a single region (although sometimes specimens were collected from a few proximate localities within region), (2) the overall pattern and degree of intraspecific variation are relatively consistent for the duration of the species, and (3) incorporating specimens from a few nearby localities does not significantly alter the overall pattern. The first assumption is difficult to test with our data and thus, it requires further investigation. We partially tested the latter two assumptions by plotting the WER of each species from different localities within Hokkaido and found that these localities are not distinguishable (Supplemental Figure). Furthermore, we presume that post-mortem transport is minimal, as suggested by the original studies from which our data was derived (Table 1) (see also Yacobucci, 2018).

Regarding the signals inferred from intraspecific variation, our analysis focused on a single conch parameter, the whorl expansion rate (WER). It is possible that intraspecific variation in one character may not fully represent the variation within an individual, and thus, evolutionary patterns may not be apparent. Nevertheless, previous studies revealed that some conch parameters in ammonoids are often intercorrelated (Monnet et al., 2015). WER is frequently linked to whether the conch is evolute or involute (Klug et al., 2015), and whether ornamentation is present or not. Therefore, we suggest that WER can provide meaningful insights into species variation and evolutionary trends.

Sexual dimorphism is recognized in some of the species we examined such as *Subprionocyclus minimus*, *Yezoites puerculus*, *Scaphites whitfieldi*, and *Hoploscaphites comprimus* (Landman, 1987; Futakami, 1990; Landman & Waagé, 1993b; Tanabe, 2022). In these species, dimorphic pairs of ammonoids share morphological traits in early ontogeny with growth patterns diverging later, leading to distinct conch shapes and likely different adult sizes. Both macroconchs and microconchs were included in the study. In the case of *Y*. *puerculus*, the morphological changes associated with the adult stage, which are referred to as the “morphogenetic countdown” (Seilacher & Gunji, 1993), are visible at a diameter of approximately 10 mm, while macroconchs exhibit these changes later in ontogeny (Fig. 4G). For other species, macroconchs and microconchs are not distinguishable. Although including dimorphic pairs could introduce bias, our focus on early ontogeny (2.5–9.5 mm), likely before the onset of the morphogenetic countdown, suggests that the inclusion of dimorphic ammonoids does not significantly affect our analysis.

### Magnitude of variation versus interrelationships of taxa

Our results reveal that the degree of intraspecific variation exhibits a pattern. While species-specific patterns of intraspecific variation are often ambiguous, more apparent patterns emerge at higher taxonomic levels. Specifically, phylloceratids (Phylloceratina) and scaphitids (Ancyloceratina) exhibit higher levels of intraspecific variation compared to gaudryceratids, tetragonitids (both Lytoceratina), desmoceratids, and collignoniceratids (both Ammonitina; Fig. 5). Within families, we found no significant differences in intraspecific variation, except between *Hypophylloceras ramosum* and *Phyllopachyceras ezoense*. Furthermore, the degree of intraspecific variation does not differ significantly within species from two different time periods in *Damesites damesi*, *Gaudryceras tenuiliratum*, and *H*. *ramosum*. To date, no comprehensive and rigorous phylogenetic tree has been constructed for Late Cretaceous ammonoids, including the taxa examined in this study. Therefore, it remains unclear to what extent the degree of intraspecific variation reflects a phylogenetic signal. Tajika et al. (2018) observed varying degrees of intraspecific variation in the whorl expansion rate (WER) and whorl width index (WWI) among geographic populations of modern *Nautilus* in the Indo-Pacific. Although it remains a topic of debate whether these geographic populations represent different species, the pattern in *Nautilus* differs in that slight environmental differences lead to varying degrees of morphological variation. Similarly, Hopkins (2011) explored the relationship between morphological intraspecific variation and phylogeny in late Cambrian trilobites, but found no correlation. The ontogenetic stages in the trilobite specimens were not stated in the paper, but it is possible that an unevenness of sample size for different ontogenetic stages may have masked the actual pattern. It should also be noted that a similar magnitude of intraspecific variation in scaphitid ammonoids of North America and Japan may not be rooted in the phylogenetic proximity. Considering that the external environment may also influence the degree of variation, the observed variation in scaphites likely reflects a combination of both phylogenetic and environmental factors. At this point, we cannot separate the two factors. Further data are needed from multiple species that occupy similar environmental conditions, as well as from later ontogenetic stages.

### Magnitude of variation versus species duration and geographic distribution

A larger geographic range generally increases a species’ chances of survival (Gaston & Blackburn, 2000; Manne et al., 1999). This pattern is evident in the fossil record of marine benthic mollusks, in which species with broader geographic distributions tend to have higher survivorship during background times, although this advantage may not hold during mass extinction events (Jablonski, 1986; Kiessling & Aberhan, 2007). For cephalopods, a group of marine nekto-benthic organisms, a wider geographic distribution likely offered some protection against mass extinction, at least temporarily (Landman et al., 2014). Our data reveal only a weak to moderate and negative correlation between the magnitude of intraspecific variation and geographic distribution (Fig. 8; Table 3). If we assume that extinction risk decreases with a broader geographic range during background times, our data suggest that higher intraspecific variation in whorl expansion rate (WER) did not enhance species survivorship. From a perspective of functional morphology, WER is linked to hydrodynamic properties. A higher WER, coupled with a larger umbilical space, is thought to increase drag (Hebdon et al., 2020; Naglik et al., 2016; Peterman et al., 2020). WER is also linked to body chamber length, which influences maneuverability, motility, and stability (Peterman & Ritterbush, 2022). During early ontogeny, ammonoid species likely led a planktic lifestyle, in which WER could have influenced dispersal capabilities (Klug, 2001; Westermann, 1996; Westermann & Tsujita, 1999). However, since our data show no positive correlation between WER variation and species range, it is likely that intraspecific variation in WER did not confer a functional advantage. Callomon (1985) examined a large sample of Jurassic Cardioceratidae and found no clear relationship between intraspecific variation and geographical dispersal or habitat shifts. Rogov (2020) showed that species within a single lineage of Jurassic Craspeditidae, even though they share similar geographic distribution, and duration, exhibit different degrees of intraspecific variation. Similarly, a lack of strong correlation between intraspecific variation and geographic distribution has also been reported in late Cambrian trilobites (Hopkins, 2011). As noted above, we treat local intraspecific variation as the taxon’s overall variability. Yet, species with broad geographic distribution are likely exposed to wider environmental gradients, and may therefore exhibit different degrees of variation. The absence of correlation between geographic distribution and variation observed here may, at least in part, stem from this limitation.

Geographic distribution and species duration are often correlated, likely because a wider geographic range facilitates gene flow and provides greater resilience to local extinction events (Hansen, 1980; Hopkins, 2011; Jablonski, 1987). Accordingly, our data also shows a positive correlation between geographic distribution and species longevity. Thus, it is not surprising that intraspecific variation and species duration in ammonoids are only weakly or barely correlated (Fig. 8). The relationship between intraspecific variation and species duration varies across different taxa. Liow (2007) found that species and genera with subspecies and subgenera exhibiting greater morphological variability tend to have greater longevity, although those species and subspecies with high variability were not investigated in that study. Conversely, Hopkins (2011) did not find a correlation between intraspecific variation and taxon duration. Since the time interval we examined did not include a major mass extinction event, we consider it as representing background extinction rate times (Song et al., 2021; Stanley, 2016). During this period, higher intraspecific variation in ammonoids in early ontogeny does not appear to have contributed to increased species longevity.

### Pattern of intraspecific variation in early ontogeny versus evolution

Using a growth vector model for gastropods, Urdy et al. (2010) demonstrated that growth rate is a critical factor influencing allometric patterns and, consequently, morphological variation. In ammonoids, three post-embryonic growth stages have been recognized: neanic, juvenile, and adult stages (Bucher et al., 1996; Klug, 2001). Transitions between these stages likely involve changes in growth patterns, and possibly growth rates. The conch diameters we examined for intraspecific variation (2.5–9.5 mm) likely encompass both the neanic and juvenile stages (Kawakami et al., 2022; Nishino et al., 2024; Takai et al., 2022). Our data reveal changes in the ontogenetic pattern of whorl expansion rate (WER) in many species (Figs. 3 and 4), which may correspond to transitions between growth stages. The timing of these transitions contributes to the observed differences in the magnitude and pattern of intraspecific variation among species. Estimating the growth rate of ammonoids is possible, but such estimates require numerous assumptions (Bucher et al., 1996), making them potentially inaccurate. In modern cephalopods, the growth rate changes with different temperatures; cephalopods grow faster with a higher temperature and possibly attain a larger adult size (Forsythe, 2004). Paleotemperature data, derived from stable oxygen isotopes in ammonoid shells, suggest temperature ranges of approximately 15–22 °C in the Campanian of Japan and 12.5–27 °C in the late Maastrichtian of South Dakota (Moriya et al., 2003; Witts et al., 2020). Although specific temperature data for Turonian ammonoids from Hokkaido is unavailable, the average global temperature during the Turonian was likely significantly higher than in the Campanian and Maastrichtian (Huber et al., 2018). Despite this, neither the pattern nor the magnitude of variation shows a clear difference between these stages (e.g., two scaphitid ammonoids from North America), suggesting that temperature was not the primary factor of forming the pattern of intraspecific variation, at least in the examined taxa.

Recent studies have identified differences in basal metabolic rates among Cretaceous ammonoid species, inferred from stable carbon isotopes in their shells (Tajika et al., 2023). These differences suggest variations in growth rates, which may have influenced the observed patterns of variation. However, basal metabolic rates for the ammonoids examined in this study have not been calculated and, thus, further study is needed. Related to basal metabolic rate, adult size varies significantly among species, with reported average conch diameters ranging from approximately 15 mm (*Yezoites puerculus*) to > 100 mm (*Subprionocyclus minimus*) (Harada & Tanabe, 2005; Tanabe, 2022). This variation in adult size likely reflects different timings of transitions between growth stages. Tajika et al. (2018) compared intraspecific variation across three different populations of the modern *Nautilus pompilius*, and found distinct ontogenetic shifts in WER and whorl width index. According to the study, shifts occur earlier in populations with smaller adult sizes. In ammonoids, similar factors such as phylogeny, habitat temperature, and food availability could have influenced the pattern of intraspecific variation to some extent.

The dendrogram generated from the ontogenetic patterns of intraspecific variation (Fig. 7) reveals two main clusters. The North American species *Hoploscaphites comprimus* and *Scaphites whitfieldi* (both Scaphitidae, Ancyloceratina) exhibit very similar patterns, forming a distinct cluster (Figs. 5 and 7). Another scaphitid group *Yezoites puerculus* from the Turonian of Japan also shares a similar pattern. Regarding abiotic factors, there were probably remarkable environmental differences between the two locations (Japan and North America) across the Pacific Ocean. As discussed above, the environment presumably also changed significantly between the Turonian and Maastrichtian with the closure of the Western Interior Seaway and decreasing temperature (Huber et al., 2018). Although it is possible that abiotic factors play a role in producing intraspecific variation, the similar ontogenetic pattern of intraspecific variation shared by the scaphitid species from presumably different environments suggests that a phylogenetic proximity may influence the pattern of intraspecific variation to a higher degree.

While scaphitid ammonoids exhibit similar ontogenetic patterns, most other species are nearly indistinguishable regarding the ontogenetic pattern of variation. The species within Ammonitina (*Damesites damesi*, *Tragodesmoceroides subcostatus*, and *Subprionocyclus minimus*), Phylloceratina (*Hypophylloceras ramosum* and *Phyllopachyceras ezoense*), and Lytoceratina (*Gaudryceras tenuiliratum*, two species of *Tetragonites*) are scattered throughout the dendrogram without clear patterns. An exception is *H*. *ramosum*, which clusters with scaphitid ammonoids. One explanation for this is that the dynamic time warping we employ accounts for the degree of variation, as well as the pattern, which likely placed *H. ramosum* in the cluster of scaphitid ammonoids. It is also likely that the ontogenetic pattern is shared in early ontogeny but diverges in later ontogeny. Establishing a robust phylogenetic tree and additional data from later stages of ontogeny may shed new light on the relationship between the pattern of variation and interrelationships of species.

We also compared the patterns within *D*. *damesi*, *H*. *ramosum*, and *G*. *tenuiliratum* from two successive stages (Santonian and Campanian). The Santonian and Campanian populations within each sample of *D*. *damesi* and *H*. *ramosum* exhibit a similar pattern, placing them in close proximity in the dendrogram (Fig. 7A). The pattern of *G. tenuiliratum* shows slight differences (Fig. 3F). Considering the slightly different ontogenetic trajectories in WER (Fig. 3E), the two populations may not represent a single species, although we cannot verify this only with WER [see Kawakami et al. (2022) for discussion]. Furthermore, there appears to be no stage-specific pattern of intraspecific variation (Fig. 7A). The patterns of intraspecific variation, geographic distribution, and species duration (Fig. 7) show limited associations. The scaphitid species exhibit small geographic distributions, resulting in a high correlation between variation patterns and geographic distribution. However, no other groups show a clear correlation. Similarly, the scaphitid species from North America share a very short longevity. Interestingly, the two phylloceratid species *H. ramosum* and *P. ezoense* exhibit broad geographic ranges and long durations, yet show highly different ontogenetic patterns of variation.

Future research would benefit from sampling multiple species that occur within well-defined stratigraphic horizons at a single locality, thereby minimizing time-averaging and other biases noted above. It would also be valuable to incorporate lineages with pronounced phenotypic plasticity such as *Neogastroplites* (Reeside & Cobban, 1960) to test if the patterns presented herein persist in taxa that exhibit higher morphological variation. Additionally, examining taxa occupying contrasting environments (e.g., oceanic vs. neritic habitats) separately may clarify how environment influences intraspecific variation.

## Conclusions

We examined the relationship between the degree and pattern of intraspecific variation, along with geographic distribution and species duration, with a focus on early ontogeny in Late Cretaceous ammonoids. Here we summarize our discoveries:

1. We found only a weak correlation between the magnitude of intraspecific variation in the whorl expansion rate (WER) and species duration. Additionally, there was a weak to moderate, negative correlation between geographic distribution and intraspecific variation. These results suggest that greater intraspecific variation in WER during early ontogeny does not necessarily correspond to longer species duration or broader geographic distribution. Lastly, there was no significant difference in the magnitude and pattern of intraspecific variation within species between two successive stages (Santonian and Campanian).
2. Similarly, the pattern of intraspecific variation in WER during early ontogeny showed no strong signal with respect to species duration or geographic distribution. However, there was a tendency for species with higher intraspecific variation to exhibit shorter durations and more limited geographic distributions. The pattern of intraspecific variation within species between two successive stages (Santonian and Campanian) was similar except in *Gaudryceras tenuiliratum*.
3. Despite the absence of a robust and up-to-date phylogenetic hypothesis for the taxa studied, some patterns are apparent. Three scaphitids—*Hoploscaphites comprimus* from the Maastrichtian, *Scaphites whitfieldi* from the Turonian (both from North America), and *Yezoites puerculus* from the Turonian of Japan—exhibited similar ontogenetic patterns and magnitudes of intraspecific variation. Nevertheless, the variation is produced by multiple factors, such as genetics, phenotypic plasticity, and adaptation, and we cannot conclude that the similarity is predominantly rooted in their proximate interrelationships. In contrast, other taxa representing Ammonitina, Lytoceratina, and Phylloceratina could not be distinguished based on the magnitude or pattern of intraspecific variation with the exception of *Hypophylloceras ramosum*, which exhibits a pattern similar to those of scaphitid species. The results imply the necessity of investigating the morphology more comprehensively for longer ontogenetic stages and establishing a robust phylogenetic tree.

## Supplementary Information

Supplementary material 1.

Supplementary material 2.

## Data Availability

No datasets were generated or analysed during the current study.
