# Supplementary figures and images for: Does intraspecific variation in juvenile Late Cretaceous ammonoids correlate with their systematic position, longevity and paleogeography?
Source: Swiss J Palaeontol. 2025 Aug 19;144(1):51. doi: 10.1186/s13358-025-00397-y (PMC12364979; doi:10.1186/s13358-025-00397-y)

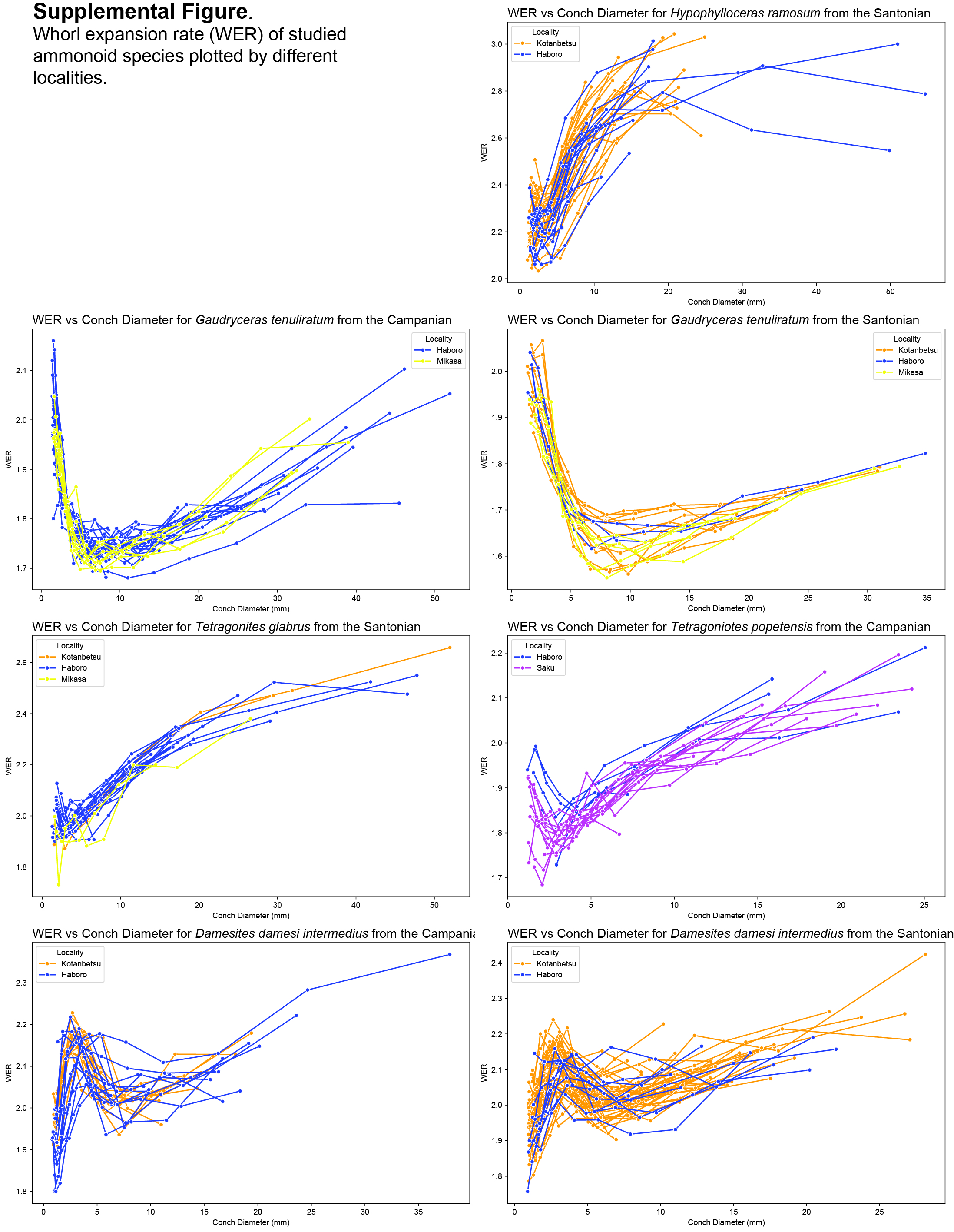

Supplement: Supplementary file 1 — Supplementary material 1. [file 13358_2025_397_MOESM1_ESM.jpg]
